# Two Practical Suggestions

**Published:** 1904-06

**Authors:** Henry Knowles

**Affiliations:** Washington, D. C.


### TWO PRACTICAL SUGGESTIONS.

By Dr. Henry Knowles, Washington, D. C.

To Make a Dentimeter.—Take the bone handle of an old mouth-mirror; break off the screw, if it is not already broken; with file or stone, flatten the end for a quarter of an inch or more; drill two small parallel holes.

To Make New Post for Richmond Crown.—When post has broken, with wheel burs and small stones, dress down remainder of post so that it is flush with cap; solder new post to a disk of platinum that approximates size of band; cut platinum so that it fits snugly inside of band; the base of post must be ground flush with cap; use minimum amount of solder in uniting new cap to band. The repaired crown simply has two caps.—*Summary.*

